# Unpacking Missionary Collections

**DOI:** 10.1080/17432200.2025.2505307

**Published:** 2025-07-11

**Authors:** Birgit Meyer, Peter Pels

**Affiliations:** a Department of Philosophy and Religious Studies, Utrecht University, The Netherlands; b Department of Cultural Anthropology and Development Sociology, Leiden University, The Netherlands

**Keywords:** Christian missions, materiality, conversion, missionary collections and exhibitions, spiritual artifacts

## Abstract

Christian missionary collections have contributed much to the development of the exhibitionary complex, but have received significantly less notice than imperial states using violence to acquire collections, and subsequent demands for restitution. This introductory essay argues that researching missionary collecting and exhibiting requires a broad approach to the materiality of collections, to recognize the multilayered biographies of artifacts, the coeval relations between missionaries and (future) converts in the mission field, as well as how archives and collections form part of technologies of empire. This “unpacking” leads to a recognition, firstly, of the salient position of religion in those processes of collecting and exhibiting; secondly, the realization that converting people was accompanied by multitemporal and multidirectional processes of converting goods; and thirdly, that despite public assertions of Christian iconoclasm, these conversions always also included turning artifacts into desecrated, secular, or even commercial goods. We conclude by arguing that these processes, including their potential reversal, need to be taken into account when considering the future of these collections.

Despite their central role in European colonial practice, Christian missionary collections and collecting have not attracted much attention in debates about colonial afterlives. “Theories of taking,” as well as the global floodlights on restitution since Emmanuel Macron’s 2017 Ouagadougou declaration, tend to focus on museums, looting, military expeditions, and negotiations between nation-states (Hicks 2020, 163; Sarr & Savoy 2018). Research into the specificities of Christian missionary collections started some twenty years ago, while the practical and discursive contribution of Christian missions to European colonial collecting and (museum) collections has only become a topic of discussion in the current decade.^1^ The research presented in this special issue highlights the central role of collecting and artifact collections in Christian mission practices. This underscores how Christianity, in its global expansion, is a profoundly material religion. While studies of conversion to Christianity have broken down the barriers between the study of religion and the social sciences since the 1990s (Comaroff and Comaroff 1991; Peel 2001; Veer 1996), little attention was paid to the relation between missions and (ethnographic) museums. Spotlighting the constitutive role of material religion at the heart of modernity’s only apparently secular(ist) exhibitionary complexes, museums, this special issue draws the study of missions out of the niche in which they have long been located.^2^ Our central aim is to showcase the centrality of missions for the making of colonial ethnological collections. The critical deconstruction of such collections from a decolonial angle needs to move beyond a taken-for-granted secular frame, acknowledging the work of missions and the trajectories of the artifacts they contributed to European museums as a central theme for research and public debate about Europe’s colonial past. It also raises questions and presents dilemmas to those who confront the indigenous spiritualities that Christians dismissed as tokens of “idolatry” (see De Witte, this issue; and the In Conversation section).

This issue is grounded in a research project entitled “Heritage and the Question of Conversion” (coordinated by us since 2021); it is devoted to missionary collecting, in the context of a larger project researching pasts, presents and futures of colonial collections in the Netherlands.^3^ Three of the four articles that follow arise from this project and research Dutch collections, while the fourth, and the essays in the In Conversation section, result from wider contacts established both before and during this project. This special issue does not leave room for a discussion of “heritage” and missions, except for noting that the relationship between tangible and intangible aspects of the past in the present is not just crucial to heritage studies but also to our concerns—not least because we are inevitably confronted with the question how temporalities of conversion were and are materially performed. We therefore “unpack” missionary collecting in this brief introduction in three steps: firstly, we consider the central and transformative role of *materiality* in the methodology and conceptualization of research into and results of the pasts of Christian missionary practices, and their collecting in particular. Secondly, we zoom in on the notion of *conversion*, since its predominantly “mentalist” conceptualization in both Christian, historical, and social scientific circles is significantly altered and expanded once we ask what must be included in its material performance, both in the colonial fields of missionization as well as for its Christian and secular audiences in Europe. Finally, we address which new departures the articles and the In Conversation section may offer when we engage with the global afterlife of Christian missionary collections, their embedding in a history of colonialism, and the life of both spirit(s) and matter, today.

## Materiality

Championed by this journal for twenty years, “material religion” appears to have a game-changing potential. Alerting scholars to the physical and corporeal dimensions of how religion is lived and tangible in the world, the material turn did not only yield due attention to religious material culture. It also prompted a critique of both the conventional text-centered focus on doctrines and beliefs within the study of religion and the secular bias in the broader social sciences and humanities through which many world-making practices of religion were overlooked. The simple insight that religion is a mundane and material matter brings into focus the phenomenon of missionary collections: they have remained relatively invisible, even though their artifacts have long provided tangible “evidence of the missionary encounter” (Dadzie, this issue) in Europe’s colonial archives. Both of us have conducted research on the work of mission societies in what is now Ghana (Meyer 1999) and Tanzania (Pels 1999) in a historical and to some extent material perspective since the 1990s. And yet, until recently we paid minimal attention to practices of missionary collecting and the concrete connections between missions and museums in which missionary collections were kept and displayed.^4^ Our current research project originated in the question: “where *are* the things that missionaries collected, after they were seen to engage in iconoclastic destruction of artifacts they classified as ‘heathen?’” The study of religion from a material angle makes us conceive of the work of missions in a fresh manner, showing work on colonial missions to be far more relevant to modern exhibitionary complexes than was assumed earlier.

The contributions to this special issue illustrate the methodological and conceptual benefits of a material approach to the making, display and circulation of missionary collections: “korwars” and numerous other artifacts collected by Protestant missionaries of the Utrecht Missionary Society (*Utrechtse Zendingsvereniging*, abbreviated herein as UZV) in Western New Guinea and displayed in various exhibitions in the late nineteenth and early twentieth century (Roussillon); artifacts taken by the Reverend Henry Townsend (Church Missionary Society) from the Yoruba city state of Abeokuta (Nigeria) to the UK in the mid-nineteenth century (Dadzie); crucifixes, rooted in the early conversion to Catholicism in the Kongo kingdom in the late fifteenth century, collected by a Dutch Catholic missionary for exhibits in the Low Countries in the 1950s (Amaral); and the trajectories of six so-called “Asante altar figures” from Ghana that were bought through the art market for and displayed in a Dutch Afrika Museum (De Witte). The four authors approach the artifacts in (former) missionary collections as an “entry point” into the wider colonial and postcolonial assemblages of humans and non-human actors situated in the historical colonial entanglements in which Europe, Africa and Papua got caught up, under highly unequal power relations. Their approach is backed by the idea that knowledge production is a material endeavor, in which artifacts operate as “epistemological mediators” (Roussillon) that are harnessed to legitimize and offer evidence of colonial-missionary categories and narratives. Unpacking missionary collections implies undertaking a micro-archaeology that excavates these categories and narratives, even when making their materialities visible requires a disturbing resuscitation of problematic terms from the colonial lexicon (such as “idol” or “fetish”) so as to move beyond them.

The question posed by Marleen de Witte “what do the life stories of the artifacts tell about religious encounters and entwinements in the larger histories of colonial and postcolonial entanglements of Africa and Europe?” aptly captures the spirit of this special issue. We study the items collected by missionaries in what is today Angola, Ghana, Nigeria and Western New Guinea not simply as instances of indigenous material cultures, but as nodes that, when interrogated patiently, reveal a so far silenced and neglected past that challenges still lingering colonial and stereotypical views of both “Europe” and its “Others.” Put in circulation by missions and rescripted with new values and meanings across their trajectory, the artifacts contain multiple temporalities. Whether kept in the museum depot or put on display, they tended to be presented in an a-historical manner as ethnographic specimens of particular ethnic groups. They were frozen in time due to subsequent ways of categorizing and representing them as evidence of “idolatry,” “ethnographica” or “(tribal) art.” Unpacking missionary collections, then, aims to mitigate against representations of the artifacts contained in them as signs of an inferior otherness that is situated in a “primitive” or “pagan” state which is deemed “backward.” Striving to undo the “denial of coevalness” (Fabian 1983) with regard to the artifacts and their original users requires scholars to track their connected biographies, step by step. This involves detailed historical and ethnographic analysis that restores the “individuals involved in their entangled trajectories as historical, political and religious subjects” (Amaral, this issue). Doing so entails accessing the history of the musealized artifacts prior to their transition from the mission field to the museum, by exploring tensions between the concerns of mission work in the field and the representation of artifacts so as to portray missionary success. This comes to the fore strongly in Ana Rita Amaral’s analysis of the use of crucifixes in northern Angola, which were seen as “fetishes” by the missionary collecting them but as indicators of African conversion by contemporary missionary exhibitions in the Vatican and Lisbon. Tracking the history of artifacts also requires paying attention to their material properties, for instance by scrutinizing them for traces of eggs, blood and other materials that indicate past practices of feeding (De Witte, this issue), as well as searching other sources for additional mediations of these artifacts, such as photographs, pamphlets or oral traditions. As Benjamina Dadzie points out, doing provenance research requires conceptualizing the colonial archive as not simply storing information, but, following Ann Stoler, acting as a “technology of empire” and hence constituting an object for research. Doing so, Dadzie works with a broad understanding of the archive as encompassing historical documents and material forms (texts, pictures, objects), thereby working across different institutions in which legacies of mission work are kept. It is through painstaking historical work, as conducted by her and the other three authors in an exemplary manner, that artifacts collected by missionaries can be made to reveal the intricacies of their trajectories from the mission fields to Europe.

It was not only missionaries who harnessed the strong material presence of korwars, swords, crucifixes and altar figures to authenticate all sorts of narratives imposed on them. Amélie Roussillon shows in detail how the “tokens of conversion,” employed in its museum and travelling exhibitions to prove how successfully the UZV fought “idolatry,” could also contribute to colonial knowledge production in general by showcasing “primitivity” and “civilizational progress” (to which the UZV proudly contributed) in secular colonial and universal exhibitions. In other words, the articles in this issue document and deconstruct step by step how artifacts got caught up in and entangled with multiple agendas. And yet, the artifacts ultimately resist being fully captured by these colonial and missionary agendas and narratives, retaining values and meanings in excess of those imposed by missions and museums across time. As “a palimpsestic accumulation of layers” (De Witte, this issue), they remain messy and unruly. By virtue of their sheer material presence, they contain the potential to subvert these layers and reveal something fresh. The detailed provenance research undertaken by the authors in this issue shows how artifacts can act as carriers and triggers of alternative memories, opening up another understanding of the past that has long been silenced in favor of dominant narratives imposed on them. Taking a material approach to missionary collections allows the artifacts therein to “mobilize memory, provoke story-telling, and cause people to act” (Rigney 2015, 18; see also Meyer 2025).

## Conversion

In order to realize how our focus on materiality transforms and expands our understanding of missionary engagements, we must, firstly, reconceptualize conversion to Christianity as multitemporal and multidirectional, rather than a linear process of individual spiritual change (cf. De Witte, this issue). Secondly, however, one should critically assess the disproportionate attention paid to the conversion of spiritual artifacts. It is one of the more important findings of the articles in this issue that, while the trope of “idolatry” centrally preoccupied missionaries when they presented their work at home, it distracted attention from the fact that the larger number of artifacts that missionaries converted into museum objects were mundane.

As to the first point: Part of their collecting practices tell us that Christian missionaries not only tried to sanctify persons or realize their full humanity by the “good news” of the Gospel (Toffa 2023), but also destroyed, desecrated or objectified—and by implication secularized—spiritual artifacts that these persons used before their conversion. The articles in this issue all show that mission practice is marked by such multiple directions of transformation, and, as a result, not only classified people as “saved” or “pagan” but also classified artifacts as “holy” or “diabolic,” or as waste, commodities, or museum “objects.”^5^ A crucial aim of this special issue is, therefore, to show that the evaluations that belong to conversion to Christianity *sensu stricto* (such as “faith” or “fetish;” see Amaral, this issue) were embedded in much broader circuits of conversion and material exchange. The trajectories of artifacts collected by Christian missionaries show that they were taken up in *new material assemblages* well beyond missionaries’ religious focus and the colonial field and often directed at quite different audiences. The *new frames of meaning* that “converted artifacts” (Thomas 1991, 151) adopted as a result both affirmed and excluded specific material aspects of conversion processes, pitting faith, superstition, science and commodification against each other—sometimes simultaneously. This also shows that the *temporalities* those artifacts were made to perform for different audiences were not linear, and the dehistoricization of these artifacts needs to be countered (Amaral, this issue; De Witte, this issue). People curating these artifacts cannot do without the different pasts of artifacts when they try to determine what futures these artifacts may have in store, as the essays in the In Conversation section demonstrate.

A provisional typology of the new material assemblages into which artifacts collected by missionaries entered includes “idolatry,” colonial ethnography, museums, and trade, all overlapping in different ways. The “idolatry” that provoked missionary iconoclasm of “pagan” imagery is more properly reconceptualized as an “iconoclash” (Latour 2002): the powerful images that people destroy or remove also generate material reminders of their destruction or desecration that conserve or sometimes even enhance their powers. The “converted artifact,” especially when presented as a trophy of successful Christian missionization in an exhibition, expresses that layered clash of images in material form. One of the important insights offered by the articles in this issue is that this materialization of the conversion frame often first occurred in the exhibitionary spectacles that missionaries staged *at home*: they show that a drum, an Esu statue, or a korwar was often first defined by *exhibitionary* discourse as a mission trophy voluntarily surrendered after conversion, and a large number of such artifacts travelled to Europe not on the ticket of “idolatry,” but, for example, as political gifts (Dadzie, this issue; Roussillon, this issue). It may not be coincidental that these examples refer to early Protestant missions, since Catholic missionaries are often described as less iconoclastic and more inclined to respect ritual materials—but this should not blind us to the fact that Catholics also destroyed “fetish huts” and engaged in spectacular displays of converted artifacts at home (Amaral, this issue; Pels 2023, ch.7).

Missionary engagements with *museums, ethnography and trade*, in contrast, show “conversions” of artifacts in a much broader sense: missionaries assembled collectibles—even when desired in the mission field itself (Dadzie, this issue; Amaral, this issue)—by a different “surgical cut” from their origins (Kirshenblatt-Gimblett 1998, 18). Colonial ethnography, especially as practiced in museums, usually alienated artifacts from their original uses by reducing them to (or fictionalizing them as) an ethnicized “other” on a lower rung in the human hierarchy (Amaral, this issue; De Witte, this issue). Moving beyond strictly speaking Christian assemblages, being identified as part of an “other’s” tradition conferred a heritage value on an artifact that could label it as the non-alienable property of a museum but that could just as easily convert it into monetary value in the rising “tribal” art market, with which Dutch Catholic missionaries engaged from the early 1950s (De Witte, this issue; Pels 2023, 193). Likewise, missionary exhibitions often added the raising of revenue to their primary propaganda purposes by converting artifacts from the mission field into commodities for sale (Roussillon, this issue; Pels 2023, ch.7).

If some meanings were inscribed on the materials themselves (as in the case of some Papuan items put on display by Dutch Protestants; Roussillon, this issue), many new meanings adopted in new material assemblages could only be articulated by acts of classification that remained relatively autonomous from and invisible on the materials themselves. In the time span (1850–1960) to which most articles refer, terms like “fetish” and “idol” were commonly used interchangeably by Christian missionaries (cf. Meyer 2024) without regard to parallel secular uses of “fetish” and “fetishism” in modern societies. The mutability of such terms vis-á-vis their material carriers becomes clear from Father Jan Vissers’ application of “fetish” to Kongo crucifixes, which were regarded, before Africa became a “Dark Continent” in the European imagination, as part of a Catholic Kongo defined as sovereign by the Vatican (Amaral, this issue). Vissers’ “demotion” of the crucifix from Christian worship to an instrument of witchcraft and devilry contrasts with some of his fellow-Spiritans at the Afrika Museum in the Netherlands, who avoided the term “fetish,” used as a sales category in the “tribal art” market, and redefined African artifacts as a religious “art” (De Witte, this issue). What is striking, however, about classifications of missionary artifacts as either “art,” “ethnography” or commodity for sale (or all at the same time), is that they perform a “cut” from relations established in the mission field similar to what the Christian language of “conversion” does, often obscuring these relations by reclassifying these artifacts.

This, finally, shows how a material approach to conversion changes its conception of time, shifting away from the “before-and-after” scheme of a successful linear achievement of personal salvation or reduction to a museum object (De Witte, this issue). Exhibiting a “converted artifact” itself continues to display its “pagan” past, to such an extent that one wonders how it can achieve its spectacular exhibitionary effects without “presencing” some of its past spiritual powers (Pels 2023, 123). But in a more general sense, the biographical approach to artifacts, and “rethinking coevalness” in the context of their collection, portray these artifacts as historically layered, multitemporal storehouses of meanings and uses (Amaral, this issue; De Witte, this issue): we may recognize that an artifact presented as a mission trophy might have been a political gift (Dadzie, this issue), or that the spiritual value once given to an African crucifix in the past contradicts the validity attributed to it by a missionary much later (Amaral, this issue); that the display of an African “altar” in a Dutch museum may in fact testify to the curators’ loss of knowledge of its previous spiritual meanings and uses (De Witte, this issue), or that what was understood as activities of collecting and exhibiting Christian conversion “directly contributed to the dissemination of colonial ideals and governing strategies, at home and in the colonies” (Roussillon, this issue; see also van Rooden 1996). The special contribution of these articles to “re-materializing” Christian conversion is that they make us realize that the values that Christian conversion claims to have achieved must be studied critically—but also require us to recognize its legacies, and ask what kind of futures they hold in store.

## New Departures

Missionary collections, whether long ago transmitted into secular ethnological and other museums (as shown by Roussillon and Dadzie; see also Bozsa 2019) or still kept by mission societies, raise the interests that we discussed above at a time when the effects of de-churching in Northern Europe are becoming ever more salient. Today, many mission congregations lack the means to maintain their collections and seek to place them elsewhere in less precarious conditions.^6^ It may seem paradoxical to call attention to the materiality of missionary collecting at a time when many collections are in demise and the artifacts contained in them may be set afloat, towards new destinations in secular museums, source communities in the Global South, the art market, or even rubbish bins. However, as this issue amply shows, a focus on Christian missions and the artifacts taken by them from the mission fields is a productive, tangible starting point for countering the tendency to exempt religion from the constitution of modern collecting practices and exhibitionary complexes, and vice versa. Religion forms an inalienable dimension of the colonial project in multiple ways, and should be a central focus of provenance research as well as discussions about the future of collections. Taking religion seriously—Christianity, indigenous ideas and practices framed as “traditional religion,” but also the “secular sacreds” of modern nation-states (Balkenhol, van den Hemel, and Stengs 2020)—allows us to develop a much clearer idea about the legacies of missionary collecting both in secular collections and independent, now increasingly defunct, mission museums.

Missionary museums and exhibitions long operated as mediators of the world beyond Europe, shaping the imaginaries of their Christian audiences through stubbornly resilient templates regarding non-Western others. Tracking missionary collections enables us to identify contemporary repercussions of past practices of Christian world-making both in Europe and the mission fields. As argued by De Witte, one legacy of mission work concerns the entanglement of “missionary work and race-making in the colonial encounter.” Especially the spiritual artifacts assembled by the mission have been read through “a mental and affective grammar of racial inequality” that still informs racialized or even racist views of Europe’s others, even though many Christian missions have long rejected this grammar in favor of aspirations to equal partnership. Moreover, aspects of this grammar prove to be resilient, as Christians, both in their home societies in the Global South and in the diaspora, mobilize them vis-à-vis these spiritual artifacts. Resuscitating old missionary semantics of “idol” or “fetish,” such artifacts may still induce anxieties in beholders who perceive them as animated and even diabolically alive, instead of being acknowledged as an important indigenous cultural heritage.

Our In Conversation section shows poignantly how museum curators grapple with artifacts which appear as spiritually charged. Once the objectification of such artifacts by secularist cataloging, storage and display is problematized as a dominant, yet not fully accomplished, museum practice, questions arise about how to deal with them, and ultimately, what their future location should be. Edith Franke and Susanne Rodemeier raise these questions with regard to “dzokawo” (“magic cords”) and an Adu Zantua figure, which Protestant missionary societies brought to the collection of the Museum of Religions, Marburg. They see museums and collections “as places of interaction and lively exchange” into which people living in the regions from which the artifacts in question hail must be included. Claudia Augustat reflects on an eye-opening visit to the storage of the Weltmuseum Wien with members of the Tukano indigenous group, in which the depot turned into an uncanny place containing dangerous matter, rather than the sanitized secular space it is usually assumed to be. These museum curators show the practical relevance of developing a perspective on artifacts as coated with layers of values and meanings that demand to be unpacked, as well as recognizing the potentialities enshrined in the indigenous ontologies and epistemologies that were overlaid with missionary and colonial ethnographic ascriptions. Doing so raises pressing questions with regard to the maintenance, display and future destination of the collections. Finally, Kodzo Gavua proposes an approach to musealized artifacts as “epistemic assets” through which we can envision new pathways for recognizing that Europe and Africa have been caught up in longstanding global entanglements. He argues that new research partnerships with various stakeholders from the Global North and South are needed to scrutinize colonial collections, so as to develop “new knowledge and narratives” that will “also foster the restoration of the dignity of Africans” and work towards restitution. This summarizes the aim of research that “unpacks” missionary collections quite precisely: by an approach that focuses on artifacts collected, we seek to contribute to the restoration of knowledge and narratives that transform both stereotypes of European expansion and ignorance or prejudice denying the full humanity of Europe’s “others”—in ways that may repair the damage done by highly unequal global relationships of power.

## Notes

1 See, for instance the works by Bozsa (2019), Cannizzo (1998), Jacobs and Wingfield (2014), Jacobs, Knowles, and Wingfield (2015), Morgan (2016), Zerbini (2021), Wates (2006), published as Kahn (2023). The Sainsbury Research Unit, Norwich, and the Center for Religious Studies (CERES) at Ruhr University Bochum form stimulating hubs for research on missionary collections. Recently, the *Arbeitskreis Missionssammlungen* (working group missionary collections) was established in Germany to bring together scholars, curators and other stakeholders who research the past and possible futures of missionary collections in Germany.

2 In this special issue, our focus is restricted to Christian missions, mostly in the period after 1800. It would be fascinating to extend discussions to collecting under the aegis of other colonial relationships and religious missions (such as those established, for example, by the Ottoman Empire) but this requires other experts and collaborations.

3 Our research project is work package 3B on “Heritage and the Question of Conversion” is situated within *Pressing Matter: Ownership, Value, and the Question of Colonial Heritage in Museums* (Dutch Science Agenda [NWA] grant, 2021–25; project leaders Susan Legene and Wayne Modest, VU University Amsterdam).

4 Pels, however, first presented work on a missionary museum at a conference in 1998 (published as Pels 2023, ch. 7).

5 Across their trajectory from mission fields to Western museums, the items collected were grouped into multiple generalizing categories, including “object.” This is not a neutral term, and in the debates about the provenance of colonial collections in which we are involved, the term object has been found problematic, as it naturalizes the objectification of material forms into the secular logic of the museum. While it is impossible to avoid the use of general, English-language terms, we take heed of this criticism, and rather use “artifact.” See de Witte & Meyer (2025).

6 Catholic congregations in the Netherlands and Germany, in particular, have sold, given away or discarded (parts of) their collections since the 1970s, or are struggling to find support for the museums they once established. The Afrika Museum in Berg en Dal, for example, to which the articles by Amaral and De Witte refer, lost what remained of its Catholic ethos (see Pels 2023, ch.7) when three ethnographic museums in the Netherlands fused in 2014, but the Dutch branch of the Spiritans was dissatisfied with the result. It successfully reclaimed its collection in 2024 and aims at a restart under its own management. The German branch of the Society of the Divine Word, while faced with the closure of many of its museums, co-organized a “laboratory” with the Global Heritage Lab at the University of Bonn in which we both participated to investigate future uses of the museum and collections at its anthropological headquarters in St. Augustin, but also maintains the 1934 display of its mission museum in Steyl with (Dutch) provincial and community support. The “White Fathers” in Cologne and Munich offered their collections to CERES, Ruhr University Bochum, where it has become a resource for research on missionary collections.
